# Supplementary material for: A multifactor coupling prediction model for the failure depth of floor rocks in fully mechanized caving mining: a numerical and in situ study
Source: R Soc Open Sci. 2019 Aug 28;6(8):190528. doi: 10.1098/rsos.190528 (PMC6731718; doi:10.1098/rsos.190528)
Supplement: Figures S1 - S4 [file rsos190528supp1.zip › Yulong Jiang_figures_ESM/Yulong Jiang_figure 2_ESM.docx]

Fig. 2 Distribution map of the damaged floor rocks along the coal seam pitch for each numerical simulation scheme

(a)scheme #1 (b)scheme #2

(c)scheme #3 (d)scheme #4

(e)scheme #5 (f)scheme #6

(g)scheme #7 (h)scheme #8

(i)scheme #9 (j)scheme #10

(k)scheme #11 (l)scheme #12

(m)scheme #13 (n)scheme #14

(o)scheme #15 (p)scheme #16
