# Supplementary material for: A multifactor coupling prediction model for the failure depth of floor rocks in fully mechanized caving mining: a numerical and in situ study
Source: R Soc Open Sci. 2019 Aug 28;6(8):190528. doi: 10.1098/rsos.190528 (PMC6731718; doi:10.1098/rsos.190528)
Supplement: Figures S1 - S4 [file rsos190528supp1.zip › Yulong Jiang_figures_ESM/Yulong Jiang_figure 3_ESM.docx]

Fig. 3 Relationship between failure depth and the impact factors

1. (b)

(c) (d)
