# Supplementary material for: A multifactor coupling prediction model for the failure depth of floor rocks in fully mechanized caving mining: a numerical and in situ study
Source: R Soc Open Sci. 2019 Aug 28;6(8):190528. doi: 10.1098/rsos.190528 (PMC6731718; doi:10.1098/rsos.190528)
Supplement: Tables S1 - S8 [file rsos190528supp2.zip › Yulong Jiang_tables_ESM/Yulong Jiang_table 1_ESM.docx]

Table 1 Lithology and mechanical parameters of the floor strata

| Borehole length/m | Rock | Tensile strength /MPa | Compressive strength /MPa | Cohesion /MPa | Poisson ratio | Elastic modulus /GPa |
| --- | --- | --- | --- | --- | --- | --- |
| — | Coal | 0.75 | 7.78 | 1.13 | 0.25 | 2.50 |
| 2.30-3.57 | Mudstone | 3.57 | 25.58 | 1.98 | 0.29 | 3.72 |
| 3.57-3.70 | Fine-grained sandstone | 6.45 | 62.47 | 2.02 | 0.27 | 11.07 |
| 3.70-4.70 | Sandy mudstone | 3.49 | 32.63 | 2.08 | 0.28 | 3.48 |
| 4.70-7.70 | Mudstone | 3.57 | 25.58 | 1.98 | 0.29 | 3.72 |
| 7.70-20.60 | Sandy mudstone | 3.49 | 32.63 | 2.08 | 0.28 | 3.48 |
| 20.60-22.50 | Sandstone | 6.45 | 42.47 | 2.17 | 0.21 | 5.72 |
