# Supplementary material for: A multifactor coupling prediction model for the failure depth of floor rocks in fully mechanized caving mining: a numerical and in situ study
Source: R Soc Open Sci. 2019 Aug 28;6(8):190528. doi: 10.1098/rsos.190528 (PMC6731718; doi:10.1098/rsos.190528)
Supplement: Tables S1 - S8 [file rsos190528supp2.zip › Yulong Jiang_tables_ESM/Yulong Jiang_table 2_ESM.docx]

Table 2 The orthogonal numerical simulation schemes

| Test number | Test schemes | | | |
| --- | --- | --- | --- | --- |
|  | Mining face length/m | Coal bed pitch/° | Burial depth/m | Aquifer water pressure/MPa |
| #1 | 80 | 0 | 350 | 0 |
| #2 | 80 | 5 | 400 | 1.5 |
| #3 | 80 | 10 | 450 | 3 |
| #4 | 80 | 15 | 500 | 4.5 |
| #5 | 120 | 0 | 400 | 3 |
| #6 | 120 | 5 | 350 | 4.5 |
| #7 | 120 | 10 | 500 | 0 |
| #8 | 120 | 15 | 450 | 1.5 |
| #9 | 150 | 0 | 450 | 4.5 |
| #10 | 150 | 5 | 500 | 3 |
| #11 | 150 | 10 | 350 | 1.5 |
| #12 | 150 | 15 | 400 | 0 |
| #13 | 180 | 0 | 500 | 1.5 |
| #14 | 180 | 5 | 450 | 0 |
| #15 | 180 | 10 | 400 | 4.5 |
| #16 | 180 | 15 | 350 | 3 |
