# Supplementary material for: A multifactor coupling prediction model for the failure depth of floor rocks in fully mechanized caving mining: a numerical and in situ study
Source: R Soc Open Sci. 2019 Aug 28;6(8):190528. doi: 10.1098/rsos.190528 (PMC6731718; doi:10.1098/rsos.190528)
Supplement: Tables S1 - S8 [file rsos190528supp2.zip › Yulong Jiang_tables_ESM/Yulong Jiang_table 3_ESM.docx]

Table 3 Failure depths of floor rocks in each numerical test

| Schemes | Failure depths | Schemes | Failure depths |
| --- | --- | --- | --- |
| #1 | 7.65 m | #9 | 12.17 m |
| #2 | 9.94 m | #10 | 19.12 m |
| #3 | 10.97 m | #11 | 18.90 m |
| #4 | 18.77 m | #12 | 18.53 m |
| #5 | 12.17 m | #13 | 12.13 m |
| #6 | 10.07 m | #14 | 18.94 m |
| #7 | 18.64 m | #15 | 18.78 m |
| #8 | 18.79 m | #16 | 18.84 m |
