# Supplementary material for: A multifactor coupling prediction model for the failure depth of floor rocks in fully mechanized caving mining: a numerical and in situ study
Source: R Soc Open Sci. 2019 Aug 28;6(8):190528. doi: 10.1098/rsos.190528 (PMC6731718; doi:10.1098/rsos.190528)
Supplement: Tables S1 - S8 [file rsos190528supp2.zip › Yulong Jiang_tables_ESM/Yulong Jiang_table 4_ESM.docx]

Table 4 Average failure depth of floor rocks at each impact factor

| Impact factor | Failure depths | Impact factor | Failure depths |
| --- | --- | --- | --- |
| mining face length of 80 m | 11.83 m | burial depth of 350 m | 13.87 m |
| mining face length of 120 m | 14.92 m | burial depth of 400 m | 14.86 m |
| mining face length of 150 m | 17.18 m | burial depth of 450 m | 15.22 m |
| mining face length of 180 m | 17.17 m | burial depth of 500 m | 17.17 m |
| coal bed pitch of 0° | 11.03 m | aquifer water pressure of 0 MPa | 15.94 m |
| coal bed pitch of 5° | 14.52 m | aquifer water pressure of 1.5 MPa | 14.94 m |
| coal bed pitch of 10° | 16.82 m | aquifer water pressure of 3.0 MPa | 15.28 m |
| coal bed pitch of 15° | 18.73 m | aquifer water pressure of 4.5 MPa | 14.95 m |
