# Supplementary material for: A multifactor coupling prediction model for the failure depth of floor rocks in fully mechanized caving mining: a numerical and in situ study
Source: R Soc Open Sci. 2019 Aug 28;6(8):190528. doi: 10.1098/rsos.190528 (PMC6731718; doi:10.1098/rsos.190528)
Supplement: Tables S1 - S8 [file rsos190528supp2.zip › Yulong Jiang_tables_ESM/Yulong Jiang_table 5_ESM.docx]

Table 5 Multiple linear regression analysis results

| Source | Sum of squares | Degree of freedom | Mean square | F value | Significance F |
| --- | --- | --- | --- | --- | --- |
| regression | 11 | 71.83756 | 50.60076 | 7.748152 | 0.003194 |
| residual | 4 | 202.403 | 6.530687 |  |  |
| total | 15 | 274.2406 |  |  |  |
