# Supplementary material for: A multifactor coupling prediction model for the failure depth of floor rocks in fully mechanized caving mining: a numerical and in situ study
Source: R Soc Open Sci. 2019 Aug 28;6(8):190528. doi: 10.1098/rsos.190528 (PMC6731718; doi:10.1098/rsos.190528)
Supplement: Tables S1 - S8 [file rsos190528supp2.zip › Yulong Jiang_tables_ESM/Yulong Jiang_table 6_ESM.docx]

Table 6 Failure and damage characteristics of the floor rocks at the burial locations of each probe sensor

| Mining face | Probe sensor | Buried depth/m | Failure or not | Mutation type | Failure depth/m |
| --- | --- | --- | --- | --- | --- |
| 100502-80 | 1 | 4.93 | Y | compressive-tensile strain mutation | 12.5-14.65 |
|  | 2 | 7.93 | Y | abnormal mutation |  |
|  | 5 | 9.5 | Y | compressive-tensile strain mutation |  |
|  | 3 | 10.93 | Y | abnormal mutation |  |
|  | 6 | 12.5 | Y | compressive-tensile strain mutation |  |
|  | 4 | 14.65 | N | --- |  |
|  | 7 | 15.5 | N | --- |  |
|  | 8 | 17.5 | N | --- |  |
|  | 9 | 19.5 | N | --- |  |
| 100502-180 | 10 | 12.2 | Y | compressive-tensile strain mutation | 17.5-19.2 |
|  | 15 | 13.5 | Y | compressive-tensile strain mutation |  |
|  | 11 | 15.2 | Y | compressive-tensile strain mutation |  |
|  | 12 | 17.2 | Probe damaged | --- |  |
|  | 16 | 17.5 | Y | compressive-tensile strain mutation |  |
|  | 13 | 19.2 | N | --- |  |
|  | 17 | 20 | N | --- |  |
|  | 14 | 22 | N | --- |  |
