# Supplementary material for: A multifactor coupling prediction model for the failure depth of floor rocks in fully mechanized caving mining: a numerical and in situ study
Source: R Soc Open Sci. 2019 Aug 28;6(8):190528. doi: 10.1098/rsos.190528 (PMC6731718; doi:10.1098/rsos.190528)
Supplement: Tables S1 - S8 [file rsos190528supp2.zip › Yulong Jiang_tables_ESM/Yulong Jiang_table 7_ESM.docx]

Table 7 Significance analysis of the impact factors on the floor rocks

| impact factor | sum of squares | degree of freedom | mean square | F |  | significance |
| --- | --- | --- | --- | --- | --- | --- |
| mining face length | 76.832075 | 3 | 25.61069167 | 1.91926772 | 3.86 | - |
| coal bed pitch | 131.7711 | 3 | 43.9237 | 3.291646343 | 3.86 | * |
| burial depth | 22.95912 | 3 | 7.65304 | 0.573519561 | 3.86 | - |
| aquifer water pressure | 2.646325 | 3 | 0.882108333 | 0.066105284 | 3.86 | - |
| error | 40.03197375 | 3 | 13.34399125 |  |  |  |

Notes: (1), and

(2) If , this impact factor is of high significance, and denoted by “**”

(3) If , this impact factor is significant, and denoted by “*”

(4) If , this impact factor is not significant and denoted by “-”.
