# Supplementary material for: A multifactor coupling prediction model for the failure depth of floor rocks in fully mechanized caving mining: a numerical and in situ study
Source: R Soc Open Sci. 2019 Aug 28;6(8):190528. doi: 10.1098/rsos.190528 (PMC6731718; doi:10.1098/rsos.190528)
Supplement: Tables S1 - S8 [file rsos190528supp2.zip › Yulong Jiang_tables_ESM/Yulong Jiang_table 8_ESM.docx]

Table 8 Range of failure depth by each impact factor

| Impact factor | Range |
| --- | --- |
| Mining face length | 5.35 m |
| Coal bed pitch | 7.70 m |
| Burial depth | 3.30 m |
| Aquifer water pressure | 1.00 m |
